# Combining gray matter volume in the cuneus and the cuneus-prefrontal connectivity may predict early relapse in abstinent alcohol-dependent patients

**DOI:** 10.1371/journal.pone.0196860

**Published:** 2018-05-07

**Authors:** Junkai Wang, Yunli Fan, Yue Dong, Mengying Ma, Yuru Dong, Yajuan Niu, Yin Jiang, Hong Wang, Zhiyan Wang, Liuzhen Wu, Hongqiang Sun, Cailian Cui

**Affiliations:** 1 Neuroscience Research Institute, Peking University, Beijing, China; 2 Department of Neurobiology, School of Basic Medical Sciences, Peking University, Beijing, China; 3 Key Laboratory of Neuroscience, The Ministry of Education and Ministry of Public Health, Beijing, China; 4 Beijing Hui-Long-Guan Hospital, Peking University, Beijing, China; 5 Department of Magnetic Resonance, General Hospital of Armed Police Forces, Beijing, China; 6 Stereotactic and Functional Neurosurgery Laboratory, Beijing Neurosurgical Institute, Capital Medical University, Beijing, China; 7 Peking University Sixth Hospital/Institute of Mental Health and Key Laboratory of Mental Health, Ministry of Health, Peking University, Beijing, China; Xuanwu Hospital, Capital Medical Universty, CHINA

## Abstract

**Background:**

Developing more effective strategies to prevent relapse remains one of the major challenges of treating substance dependence. Previous studies have identified brain abnormalities in abstinent alcoholics. However, whether these persistent brain deficits in abstinence could predict early relapse to alcohol use has not been well established. This study aimed to identify biomarkers of relapse vulnerability by investigating persistent brain abnormalities in abstinent alcohol-dependent patients.

**Methods:**

Brain imaging and impulsive behavior data were collected from 56 abstinent alcohol-dependent male inpatients and 33 age-matched male healthy controls. Voxel-based morphometry was used to investigate the differences of grey matter volume between the groups. The resting-state functional connectivity was examined using brain areas with gray matter deficits as seed regions. A preliminary prospective study design was used to classify patients into abstainers and relapsers after a 62-day average abstinence period.

**Results:**

Compared with healthy controls, both relapsers and abstainers exhibited significantly reduced gray matter volume in the cuneus. Functional connectivity analysis revealed that relapsers relative to abstainers demonstrated increased cuneus-centered negative functional connectivity within a network of brain regions which are involved in executive control and salience. Abnormal gray matter volume in the left cuneus and the functional connectivity between the right cuneus and bilateral dorsolateral prefrontal cortex could successfully predict relapse during the 3-month follow-up period.

**Conclusions:**

Findings suggest that the abnormal gray matter volume in the cuneus and resting-state cuneus-prefrontal functional connectivity may play an important role in poor treatment outcomes in alcoholics and serve as useful neural markers of relapse vulnerability.

## Introduction

Alcohol dependence is a chronic brain disorder characterized by poor treatment retention and high relapse rates, which causes a huge burden in societies [1]. Although efficacious treatment options are available in initiating abstinence, more than two thirds of individuals with alcohol use disorders return to social drinking within weeks or months of abstinence from alcohol [2]. So, it remains one of the major challenges for clinicians and researchers to develop more effective strategies for intervention and prevention that can achieve sustained remission from alcohol dependence. There is evidence suggesting that neurobiological factors are crucially involved in maintenance of abstinence from alcohol, which arouses great interests in identifying neurobiological markers for evaluating relapse risk by using neuroimaging as a potential tool [3].

There is an increasing body of evidence that indicates the importance of examining resting-state functional connectivity (rsFC) in various psychiatric disorders such as substance dependence [4–7]. By examining spontaneous low-frequency temporal blood oxygen level-dependent (BOLD) fluctuations, rsFC has emerged as a non-invasive and systematic approach for the exploration of large-scale networks and their interactions in healthy subjects and become a powerful tool to identify potential neurobiological markers of clinical disorders, including substance dependence [3, 8–10]. Disrupted neural activity during resting state in individuals with alcohol dependence may influence their cognitive ability to cope with high-risk situations that could trigger a relapse. To date, relatively few studies have investigated whether the rsFC differences in individuals during early alcohol abstinence could predict subsequent relapse. A previous study reported that lower resting-state synchrony within both the reward and executive control networks as well as within the visual network during early alcohol abstinence may predict subsequent relapse [11]. However, the comorbidity with other substance use and psychiatric disorders observed in this study may affect the results. In addition, some previous studies have demonstrated that gray matter (GM) structure components are directly associated with functional components [12–14]. It is likely that the rsFC differences would correspond to the alterations in grey matter volume (GMV) that are caused by long-term alcohol use. Moreover, a previous neuroimaging study has identified that GMV deficits in posterior region surrounding the parietal-occipital sulcus (including the cuneus and the precuneus) are predictive of an earlier relapse to alcohol use [15]. Taken together, these findings suggest that alterations in brain structure and their potential downstream effects in functional connectivity during rest may predict early relapse in alcohol-dependent patients (ADPs). However, no previous study has assessed how GMV deficits and their corresponding function at rest during early abstinence from alcohol dependence are associated with subsequent relapse.

Additionally, there are some clinical studies indicating that impulsivity might be causally linked to several distinct processes in drug addiction, including the drug relapse [16]. We also found that ADPs during early abstinence displayed higher impulsive behavior that may be due to GM deficits in the mesocorticolimbic system and abnormal rsFC of the reward network in a recent study by our group [17]. Therefore, it is reasonable to investigate the relationship between impulsivity and brain deficits which might predict relapse vulnerability. Based on the relationships, we could speculate the role of impulsivity in vulnerability to relapse and utilize these findings to develop more effective strategies for rehabilitation.

The current study was designed to investigate structural and resting-state functional brain abnormalities in ADPs receiving inpatient treatment that subsequently abstained or relapsed, as compared with healthy controls (HCs) and the possible role of impulsivity in the relapse process. Behavioral impulsivity tasks and magnetic resonance imaging (MRI) scans were first performed in all participants after inpatient treatment. All ADPs were then followed up for a period of 3 months after discharge from inpatient treatment to access relapse outcomes. We employed voxel-based morphometry (VBM) to identify the possible GMV differences between relapsers, abstainers and HCs. Brain areas with significant differences were then used as seed regions for rsFC analysis to determine potential downstream effects in functional connectivity. Furthermore, abnormal GMV and rsFC between groups were examined for their association with the impulsivity measured using Barratt impulsiveness scale (BIS) and balloon analogue risk task (BART). Based on previous researches, we hypothesized that GM deficits and their potential downstream effects in rsFC during early stage of abstinence in ADPs would be predictive of subsequent relapse. We also expected aforementioned findings would be linked to impulsivity.

## Materials and methods

### Participants

All right-handed male patients were recruited from the alcohol rehab center in Beijing Hui-Long-Guan hospital. After enrolled in the experiment, ADPs were required to have at least 1 month (mean: 50.58 days) of abstinence and be free of psychoactive medications for at least 1 week before behavioral tests and MRI scans (more details see S1 Text). All age-matched male HCs were recruited from the local community. Each participant participated in behavioral tests and MRI scans in two separate days within 1 week. A total of 56 abstinent ADPs and 33 HCs were recruited to participate in behavioral tests and interviews. From the 89 total participants, 8 didn’t have functional MRI (fMRI) data for the following reasons: 3 patients and 1 healthy subject failed to show up for the scanning session and refused MRI screening for personal reasons, 3 patients and 1 healthy subject had excessive head motion during resting fMRI scan resulting in invalid data. As a consequence, the resting fMRI data were available for 50 ADPs and 31 HCs. All participants received financial compensation for their participation. The experimental procedure was approved by the research ethics committee of Beijing Hui-Long-Guan hospital. Written informed consent was obtained from all participants after the study had been fully explained.

All ADPs were diagnosed as alcohol dependence according to DSM-IV criteria by experienced psychiatrists. A Chinese version mini international neuropsychiatric interview was performed to exclude other Axis I psychiatric disorders in all subjects [18]. Michigan alcohol screening test (MAST [19]) was used to assess the severity of alcoholism. Additionally, current status of anxiety and depression in ADPs were assessed using the Hamilton anxiety scale (HAMA [20]) and the Hamilton depressive scale (HAMD [21]). ADPs had no previous substance dependence or current substance abuse other than alcohol and nicotine. Additional exclusion criteria for all participants included: 1) A history of head trauma or cranial surgery; 2) The presence of any past or current neurological disease; 3) Current or lifetime history of stroke, hypertension and type 2 diabetes required medical intervention; 4) Age above 60 years and below 19 years. ADPs were interviewed and asked whether they abstained or relapsed by telephone once every two weeks for a period of 3 months after an average of 62 days abstinent (SD = 17.00). According to the results of follow-up, a total of 56 ADPs were classified into abstainers or relapsers. During the follow-up, ADPs were considered to have relapsed if they had consumed at least one drink (a drink contains 13.6 grams of pure alcohol). 35 subjects reported to have relapsed and 21 reported to have remained abstinent from alcohol (The relapse rate was 62.5%). Demographic, clinical and behavioral data were analyzed with SPSS 11.5 using one-way ANOVA, 2-sample *t* tests or Chi-square test. All results were summarized in Table 1.

**Table 1:** Demographics and behavioral assessments of participants.

Characteristic	Relapsers	Abstainers	Healthy controls	*P* Value
Participants (n)	35 males	21 males	33 males	─
Age, mean (SD), y	41.80 (9.53)	45.95 (7.07)	42.88 (6.05)	0.15
Education ^**b**^, mean (SD), y	11.43 (2.77)	10.62 (3.46)	9.09 (3.48)	**0.013**
Age of onset of drinking, y	19.03 (3.19)	20.81 (3.59)	─	0.059
Average dose of lifetime ^**b**^^,^ ^**c**^ (no. of drinks^d^/d)	12.41 (4.76)	12.04 (4.83)	0.36 (0.49)	**< 0.001**
Dose during peak use ^**b**^^,^ ^**c**^ (no. of drinks^d^/d)	19.32 (6.71)	17.65 (8.17)	0.36 (0.49)	**< 0.001**
Length of abstinence until MRI session, d	42.38 (13.07)	42.57 (9.12)	─	0.95
Smokers ^**b**^^,^ ^**c**^, No. (%)	31 (88.57)	20 (95.24)	21 (63.64)	**0.005**
Current cigarettes/d ^**b**^^,^ ^**c**^, mean (SD), No.	19.60 (11.80)	19.71 (13.48)	11.48 (12.43)	**0.014**
Duration of smoking ^**b**^^,^ ^**c**^, mean (SD), y	20.80 (11.74)	25.90 (9.62)	14.85 (12.37)	**0.003**
MAST scores ^**a**^^,^ ^**b**^^,^ ^**c**^, mean (SD)	33.46 (4.55)	28.57 (5.02)	1.09 (1.70)	**< 0.001**
HAMA scores, mean (SD)	2.97 (2.44)	3.05 (2.29)	─	0.91
HAMD scores, mean (SD)	3.09 (2.66)	2.71 (2.45)	─	0.61
Barratt impulsiveness scale				
BIS-11 total scores^**c**^, mean (SD)	64.57 (9.61)	61.90 (6.71)	59.12 (6.59)	**0.022**
BIS-11 Nonplanning scores, mean (SD)	27.40 (4.53)	26.90 (4.65)	26.21 (3.63)	0.52
BIS-11 Motor scores, mean (SD)	21.83 (4.20)	21.62 (3.46)	20.73 (3.24)	0.45
BIS-11 Attention scores ^**a**^^,^ ^**c**^, mean (SD)	15.34 (3.40)	13.38 (2.82)	12.18 (1.96)	**< 0.001**
Balloon analogue risk task				
Average adjusted pumps ^**c**^, mean (SD), No.	49.16 (16.91)	42.88 (14.64)	37.36 (19.19)	**0.026**

Note: One-way ANOVA, t-test and Chi-square test were applied to test for group differences; statistical significance level was set at *P* < 0.05 (two-tailed). Significant *P*-values were in bold.

^**a**^ *P* < 0.05, difference between relapsers and abstainers (one-way ANOVA with Bonferroni multiple comparison test)

^**b**^ *P* < 0.05, difference between relapsers and healthy controls (one-way ANOVA with Bonferroni multiple comparison test)

^**c**^ *P* < 0.05, difference between abstainers and healthy controls (one-way ANOVA with Bonferroni multiple comparison test)

^**d**^ A drink contains 13.6 grams of pure alcohol. Abbreviations: MAST, Michigan Alcoholism Screening Test (Selzer ML 1971); BIS-11, Barratt Impulsiveness Scale 11th version (Patton JH et al. 1995); HAMA, Hamilton Anxiety Scale (Hamilton M 1959); HAMD, Hamilton Depressive Scale (Hamilton M 1960); MRI, magnetic resonance imaging

### Assessment of impulsivity

#### Barratt impulsiveness scale

Impulsivity in all subjects was assessed by the Chinese version of the Barratt impulsiveness Scale, 11th edition (BIS-11), a validated questionnaire that measures impulsiveness in three main dimensions (attention, motor and non-planning impulsiveness) [22]. This scale contains thirty 4-point Likert-type items. Items are rated from 1 (rarely/never) to 4 (almost always/always) and higher scores represent higher impulsivity. The BIS scores of subjects were listed in Table 1.

#### Balloon analogue risk task (BART)

A computerized version of balloon analogue risk task [23], as described in our previous study [17] was programmed by using E-Prime v1.1 experiment generation software (Psychology Software Tools, Inc., Pittsburgh, PA, USA). Briefly, subjects were told they would be presented with 30 virtual balloons on the computer screen. The participant was free to choose either inflating a balloon to increase earnings (¥0.05/pump) by clicking the left mouse button or stopping pumping to retain accumulated earnings at any point before the balloon burst by clicking the right mouse button. Each balloon was set to burst after an unpredictable number of pumps and at this point all the accumulated earnings would be lost. Participants were encouraged to make as much money as possible. The dependent measure of this task was the average number of pumps on all unexploded balloon trials during the performance (average adjusted pumps) and higher scores represented higher impulsivity [23]. One relapser and one HC did not complete the task and two abstainers were excluded from analysis because their scores were more than two standard deviations away from the mean.

### MRI data acquisition

All imaging acquisitions were similar to our previous study [17]. Neuroimaging procedures were conducted on a Siemens Tim Trio 3.0 T scanner (Siemens Medical Solutions, Erlangen, Germany). Participants were instructed to keep their head still in the scanner with their eyes closed and to stay awake (confirmed by themselves after the scanning). Earplugs and a head coil with foam pads were used to minimize scanner noise. Whole brain high-resolution T1-weighted images were acquired by using a multiecho magnetization prepared rapid gradient echo (MPRAGE) sequence. The acquisition parameters were as follows: repetition time = 2300ms, echo time = 2.98ms, flip angle = 9°, acquisition matrix = 240 × 256, field of view = 256 mm ×256 mm, 176 slices, voxel size = 1 ×1 × 1 mm^3^. BOLD functional imaging was conducted using a T2*-weighted single-shot, gradient-recalled echo planar imaging (EPI) sequence. Scanning parameters were: repetition time = 2000 ms, echo time = 30 ms, flip angle = 90°, acquisition matrix = 64 × 64, field of view = 256 mm × 256 mm, slice thickness = 4 mm, gap = 1 mm, voxel size = 3.91 × 3.91 × 5 mm^3^, 29 axial slices, 180 image volumes.

### Data processing and analysis

VBM technique was used to investigate whole-brain GMV abnormalities in relapsers and abstainers. High-resolution T1 images were processed by using SPM8 (Wellcome Department of Imaging Neuroscience, London, UK; http://www.fil.ion.ucl.ac.uk/spm). Briefly, the T1 images were first checked for visible quality issues and one abstainer was excluded due to severe artifacts. Then, MR images were segmented into GM, white matter (WM) and cerebrospinal fluid (CSF) partitions. Subsequently, the GM and WM partitions of each subject in the native space were high dimensionally registered and normalized to the standard Montreal Neurological Institute(MNI) space using diffeomorphic anatomical registration through exponentiated lie algebra (DARTEL) normalization as implemented in the SPM8. This improved method can achieve intersubject coregistration of brain images more accurately. After normalization, the images with modulation were smoothed with a Gaussian filter of 8 mm full-width half-maximum kernel. Next, we conducted voxel-by-voxel comparisons of GMV between relapsers, abstainers and HCs using a one-way ANOVA test with age, years of education, current cigarettes per day and total brain GMV as covariates. To alleviate possible edge effects between different tissues, voxels with GMV below 0.1 were excluded from the analysis. A voxel level threshold was set at *P* < 0.05 (FWE-corrected). For further exploring the relationships between abnormal GMV and behavioral measures, 10-mm sphere masks were defined around the peak voxels of the significant GM clusters. The relevant ROI signals were extracted using REST version 1.8 [24]; http://restfmri.net/forum/REST_V1.8).

**Fig 1:**
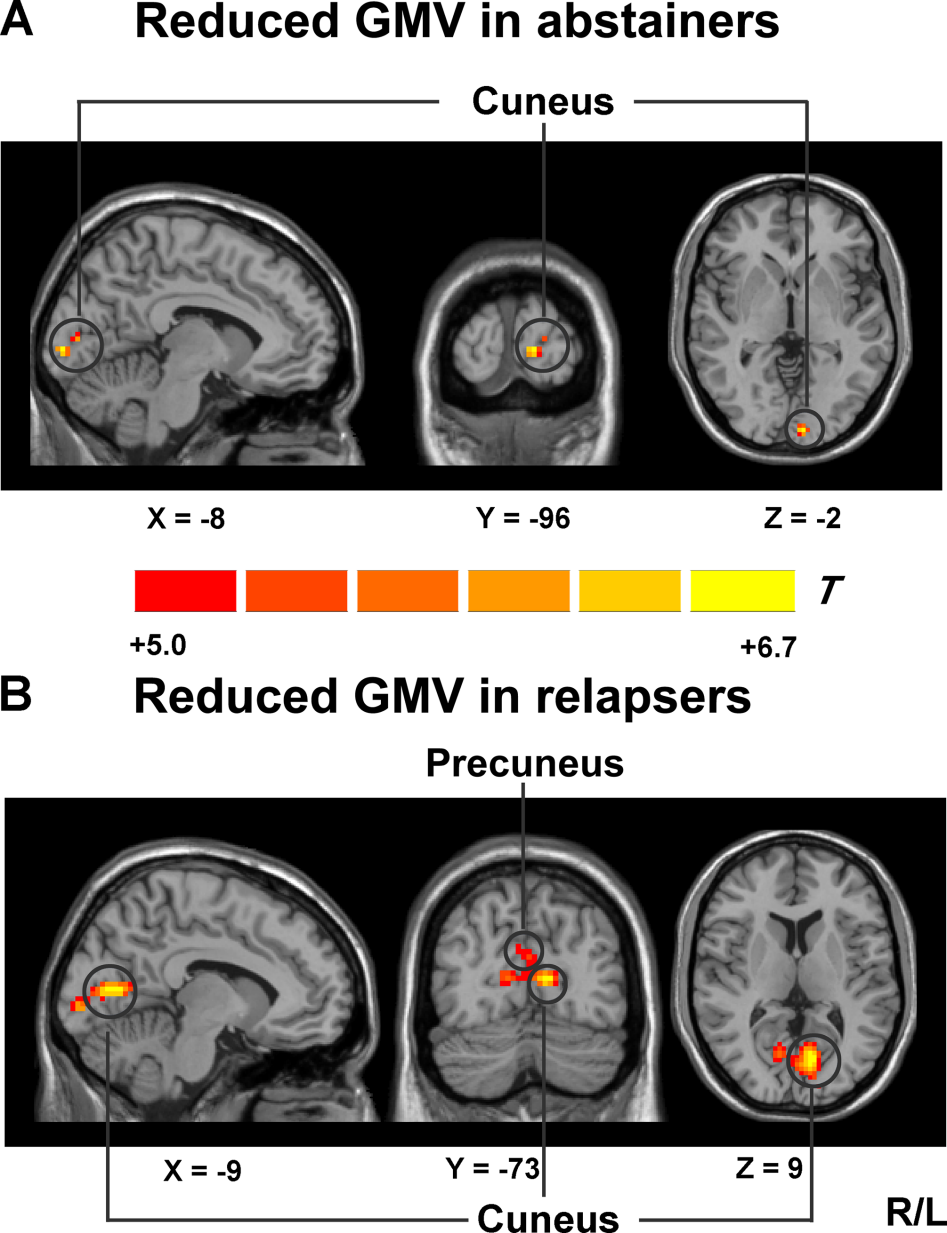
Whole-brain GMV was compared between abstainers, relapsers and HCs using SPM8 plus DAREL analysis. (A) VBM differences reflect decreased GMV in the left cuneus in abstainers compared with HCs; (B) VBM differences reflect decreased GMV in the bilateral cuneus and the right precuneus in relapsers compared with HCs. One-way ANOVA was applied to test for group differences. A voxel level threshold was set at *P* < 0.05 (FWE-corrected). Abbreviations: GMV, grey matter volume.

Preprocessing and statistical analysis of resting state fMRI (rsfMRI) images were processed using SPM8 and REST version 1.8 implemented in Matlab 7.11 (MathWorks Inc., Natick, MA, USA). Prior to pre-processing, the first 10 volumes were discarded to allow for signal stabilization. The remaining images were corrected for differences in slice acquisition time and were realigned to the first volume to reduce the confound of head motion. Participants whose head motion exceeding 2 mm in translation or 2° in rotation were excluded (3 abstainers and 1 healthy subject were excluded due to this reason). Then, the data were normalized into MNI space at an isometric voxel size of 3 × 3 × 3 mm^3^, and were smoothed by a Gaussian filter with a full width half maximum (FWHM) of 6 mm. Subsequently, the data were temporally filtered with a band-pass filter (0.01–0.08 Hz), followed by removal of the linear trend. Nine covariates of no interest were regressed from the data including six rigid motion parameters, mean WM signal, mean CSF signal and mean global signal. Based on our VBM analysis, 7 spherical seeds as the center of the regions of interest (ROIs) (radius = 6 mm) were defined for rsFC analysis, including the bilateral cuneus (MNI coordinates: -8, -96, -2 and 12, -93, 6), the right precuneus (MNI coordinates: 6, -67, 22), the right dorsolateral prefrontal cortex (dlPFC) (MNI coordinates: 2, 17, 48), the right dorsal posterior cingulate cortex (dPCC) (MNI coordinates: 0, -19, 48) and the bilateral thalamus (MNI coordinates: -18, -30, -2 and 15, -34, 1) (see Fig 1 and S1 Fig). The cross-correlation coefficients between these seed voxels and all other voxels were calculated to generate correlation maps. These correlation maps were converted to Z-value maps using Fisher’s r-to-z transformation to improve the normality of the correlation coefficients. Next, group analyses were performed for the correlation maps of each seed region. A Voxel-wise one-way ANOVA was performed to compare the correlation maps derived from each seed between relapsers, abstainers and HCs. Significance level was set at cluster level FDR corrected threshold of *P* < 0.05.

Logistic regression analysis was performed to investigate whether MRI measures, behavioral measures or the combination of MRI measures and behavioral measures predicted early relapse. First, we used binary logistic regression analysis with MRI measures or behavioral measures as predictors and relapse as the dependent variable. MRI measures included abnormal GMV and rsFC in relapsers and abstainers. Behavioral measures included demographic, clinical characteristics and behavioral assessments from Table 1. According to this analysis, we were able to assess the predictive strength of only MRI measures or behavioral measures in predicting subsequent abstinence versus relapse. Secondly, we used binary logistic regression analysis with the combination of MRI measures and behavioral measures as predictors and relapse as the dependent variable. This analysis allowed us to assess the combined predictive strength of MRI measures and behavioral measures in predicting subsequent abstinence versus relapse. Furthermore, Pearson’s correlations were used to investigate the relationships among aberrant GMV, functional connectivity and impulsive index in relapsers and abstainers separately. To determine correlation significance, *p*-value was adjusted to correct for multiple comparisons (BIS-11 total scores and average adjusted pumps) with a Bonferroni correction (2 impulsive measures = 0.05/2 = 0.025).

## Results

### Demographic data and behavioral assessments

Demographic characteristics and behavioral assessments were listed in Table 1. Relapsers, abstainers and HCs were well matched for age but years of education (F_2_,_86_ = 4.59, *P* = 0.013) was significantly longer in relapsers (*P* = 0.01) relative to HCs. The average amount of alcohol consumed per day (F_2_,_86_ = 101.86, *P* < 0.001) and the maximum number of drinks per day (F_2_,_86_ = 105.09, *P* < 0.001) were significantly greater in relapsers (*P* < 0.001 for both indicators) and abstainers (*P* < 0.001 for both indicators) relative to HCs. There were more smokers (χ^2^ = 10.3, *P* = 0.005) in relapsers (*P* = 0.015) and abstainers (*P* = 0.008) relative to HCs. Current cigarettes per day (F_2_,_86_ = 4.47, *P* = 0.014) and duration of smoking (F_2_,_86_ = 6.12, *P* = 0.003) were also significantly greater in relapsers (*P* = 0.009 for cigarettes per day; *P* = 0.036 for duration) and abstainers (*P* = 0.02 for cigarettes per day; *P* = 0.001 for duration) relative to HCs.

Impulsivity was significantly higher as reflected by BIS-11 total scores (F_2,86_ = 3.99, *P* = 0.022) in relapsers (*P* = 0.006) relative to HCs and attentional impulsivity scores (F_2,86_ = 11.02, *P* < 0.001) in relapsers (*P* < 0.001 relative to HCs; *P* = 0.013 relative to abstainers). Abstainers and HCs did not show significant difference in BIS-11 total scores and 3 subscales scores. In the BART performance, average adjusted pumps (F_2,82_ = 3.82, *P* = 0.026) were significantly higher in relapsers (*P* = 0.007) relative to HCs and there was still no significant difference between abstainers and HCs. In addition, relapsers and abstainers both showed more severe alcohol dependence as reflected by MAST scores (F_2,86_ = 651.42, *P* < 0.001) relative to HCs. There were no significant group differences between relapsers and abstainers in the scores of HAMA and HAMD.

### GMV alterations in relapsers and abstainers

#### Reduced GMV in abstainers

VBM analysis revealed that, relative to HCs, abstainers only showed significantly reduced GMV in the left cuneus (peak MNI coordinate: -8, -96, -2; cluster size = 128 voxels) (shown in S1 Table and Fig 1).

#### Reduced GMV in relapsers

Relative to HCs, relapsers also showed the most significantly reduced GMV in an area surrounding the parieto-occipital sulcus, which included the left cuneus extending to the right precuneus (peak MNI coordinate: -9, -73, 9; cluster size = 2137 voxels) and the right cuneus (peak MNI coordinate: 12, -93, 6; cluster size = 191 voxels) (shown in S1 Table and Fig 1). Moreover, Relapsers showed significantly reduced GMV in the left primary motor cortex (peak MNI coordinate: -45, -19, 37; cluster size = 362 voxels), the right dPCC (peak MNI coordinate: 0, -19, 48; cluster size = 331 voxels), the right dlPFC (peak MNI coordinate: 2, 17, 48; cluster size = 69 voxels), the right premotor cortex (peak MNI coordinate: 0, -9, 55; cluster size = 331 voxels), the bilateral thalamus (peak MNI coordinate: -18, -30, -2; cluster size = 82 voxels and peak MNI coordinate: 15, -34, 1; cluster size = 61 voxels) and the right cerebellum (peak MNI coordinate: 3, -37, -17; cluster size = 60 voxels) (see S2 Table and S1 Fig).There were no significant group differences between relapsers and abstainers in GMV and no significant increases in GMV in relapsers and abstainers relative to HCs.

### rsFC alterations in relapsers and abstainers

**Fig 2:**
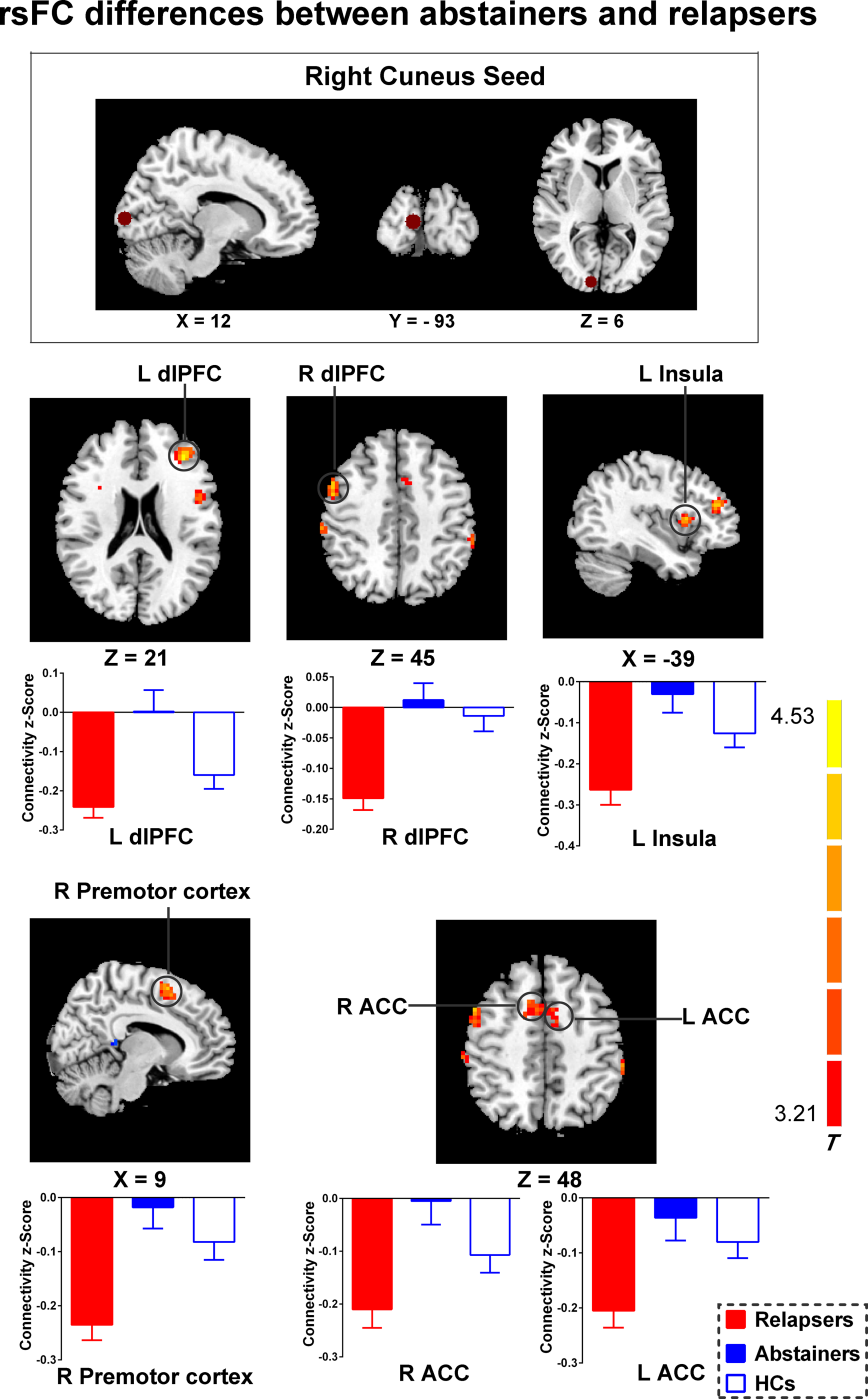
Group effects on the rsFC in the cuneus functional networks. Relative to abstainers, relapsers showed significantly increased negative functional connectivity between the right cuneus and several regions, including the the bilateral dlPFC, the bilateral ACC, the left insula and the right premotor cortex. Brain maps of representative slices of the cuneus ROI are also shown in the figure and colored dots represent seed locations. Bar graphs display mean rsFC z scores for abstainers, relapsers and HCs and the error bars represent standard deviation. Abbreviations: dlPFC, dorsolateral prefrontal cortex; ACC, anterior cingulate cortex; HCs, healthy controls; rsFC, resting-state functional connectivity.

**Fig 3:**
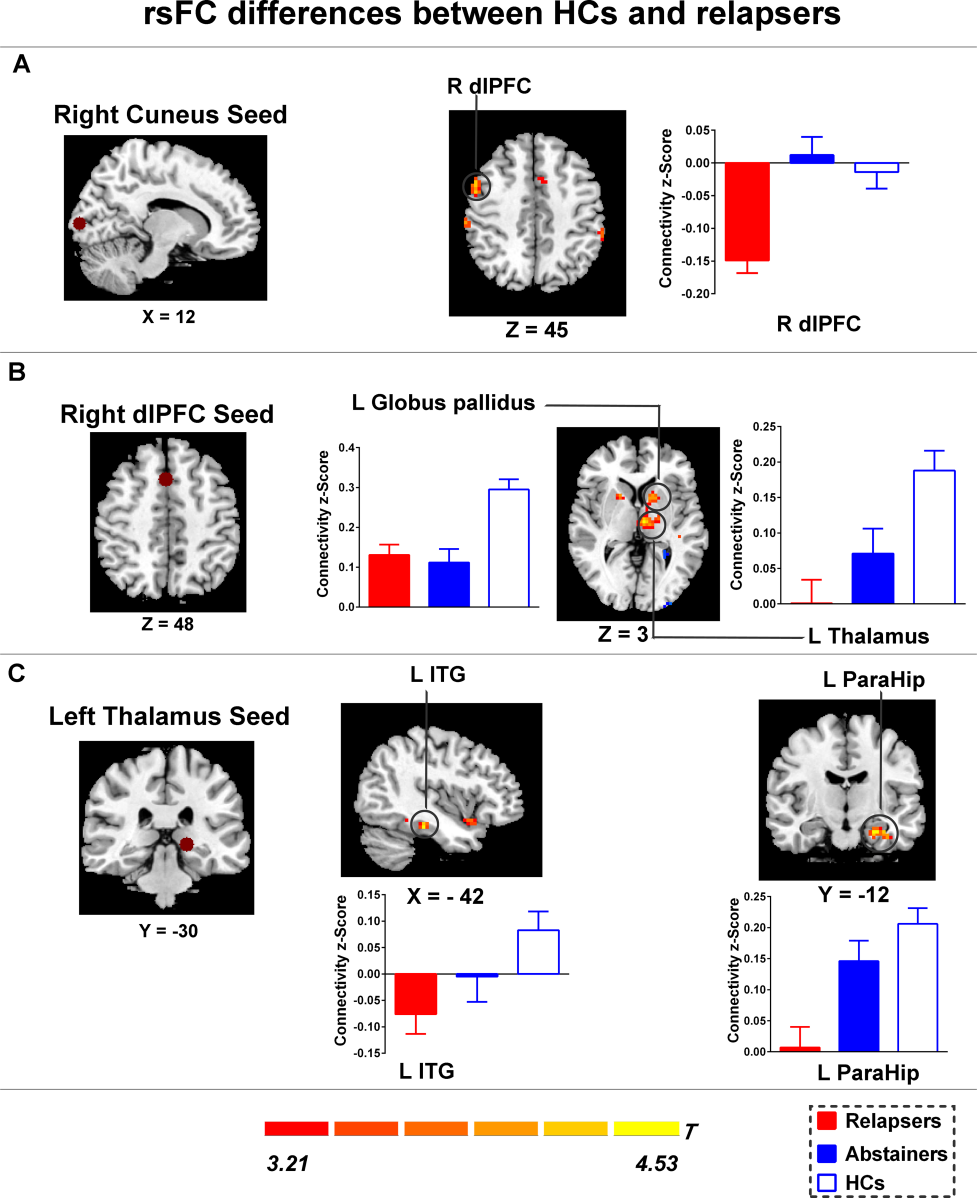
Group differences of rsFC existed between the seeds and the cluster location. (A) Compared with HCs, Relapsers showed significantly increased negative functional connectivity between the right cuneus and the right dlPFC; (B) Compared with HCs, Relapsers showed decreased rsFC between the right dlPFC and the left thalamus, and the left globus pallidus; (C) Compared with HCs, Relapsers showed decreased rsFC between the left thalamus and the left inferior temporal gyrus, and the parahippocampal gyrus. Brain maps of representative slices of ROIs are also showed in the figure and colored dots represent seed locations. Bar graphs display mean rsFC z scores for abstainers, relapsers and HCs and the error bars represent standard deviation. Abbreviations: dlPFC, dorsolateral prefrontal cortex; ITG, inferior temporal gyrus; ParaHip, parahippocampal gyrus; HCs, healthy controls; rsFC, resting-state functional connectivity.

After ROIs were selected based on our VBM results, the rsFC analysis was then conducted to characterize the ROI-based functional connectivity. In general, abstainers had significantly decreased negative functional connectivity strengths than relapsers between the right cuneus and brain regions (shown in Fig 2). Moreover, relapsers also showed reduced positive functional connectivity strengths of the dlPFC- and thalamus-related networks relative to HCs (Fig 3). There were no significant group differences between abstainers and HCs in functional connectivity strength.

#### rsFC differences between abstainers and relapsers

Specifically, relative to relapsers, abstainers demonstrated significantly decreased negative functional connectivity between the right cuneus and several regions, including the bilateral dlPFC, left insula, right premotor cortex and bilateral anterior cingulate cortex (ACC) (shown in Fig 2 and S1 Table).

#### rsFC differences between HCs and relapsers

Relative to relapsers, HCs also showed significantly decreased negative functional connectivity between the right cuneus and the right dlPFC (shown in S1 Table and Fig 3). Additionally, relapsers exhibited significantly decreased positive functional connectivity between the left thalamus seed to the left inferior temporal gyrus and to the left parahippocampal gyrus and between the right dlPFC seed to the left thalamus and to the left globus pallidus relative to HCs (shown in S1 Table and Fig 3).

### Logistic regression results

We further analyzed the predictive power of MRI measures and behavioral measures on early relapse in ADPs by creating a binary logistic regression model and defining the dependent variable as relapse or abstinence. When examining MRI measures as predictors alone, the analysis results showed that the GMV of left cuneus and the rsFC between the right cuneus and the bilateral dlPFC were statistically significant predictors (see Table 2A). The model containing all 3 variables could accurately predict group membership 94% of the time, compared with 68% accuracy in a null model (see Table 2B). When examining behavioral measures as predictors alone, the analysis results showed that there was no variable that was found to be statistically significant (see S3 Table). When examining the combination of MRI measures and behavioral measures as predictors, the results did not improve compared to using the MRI measures alone.

**Table 2:** Summary of logistic regression analysis for different MRI measure used as a predictor of early relapse.

A MRI measures as outcome predictors				
Predictor	B	SE B	Wald	*P* value
left Cuneus volume	-36.13	14.89	5.89	**0.015**
rsFC between right Cuneus and left dlPFC	11.81	6.15	3.69	**0.055**
rsFC between right Cuneus and right dlPFC	21.86	10.91	4.01	**0.045**

B The classification table of a model containing variables representing MRI measures			
Observed outcome	Predicted outcome		Percentage correct
	Relapse	Abstinence	
Relapse	32	2	94.1
Abstinence	1	15	93.8
Overall accuracy percentage of MRI index			94.0

Note: Model coded 0 for Relapsers and 1 for Abstainers. Abbreviations: rsFC, resting state functional connectivity; B, raw Beta coefficient; SE B, standard error for raw Beta coefficient.

#### Correlations between MRI measures and impulsivity in relapsers

**Fig 4:**
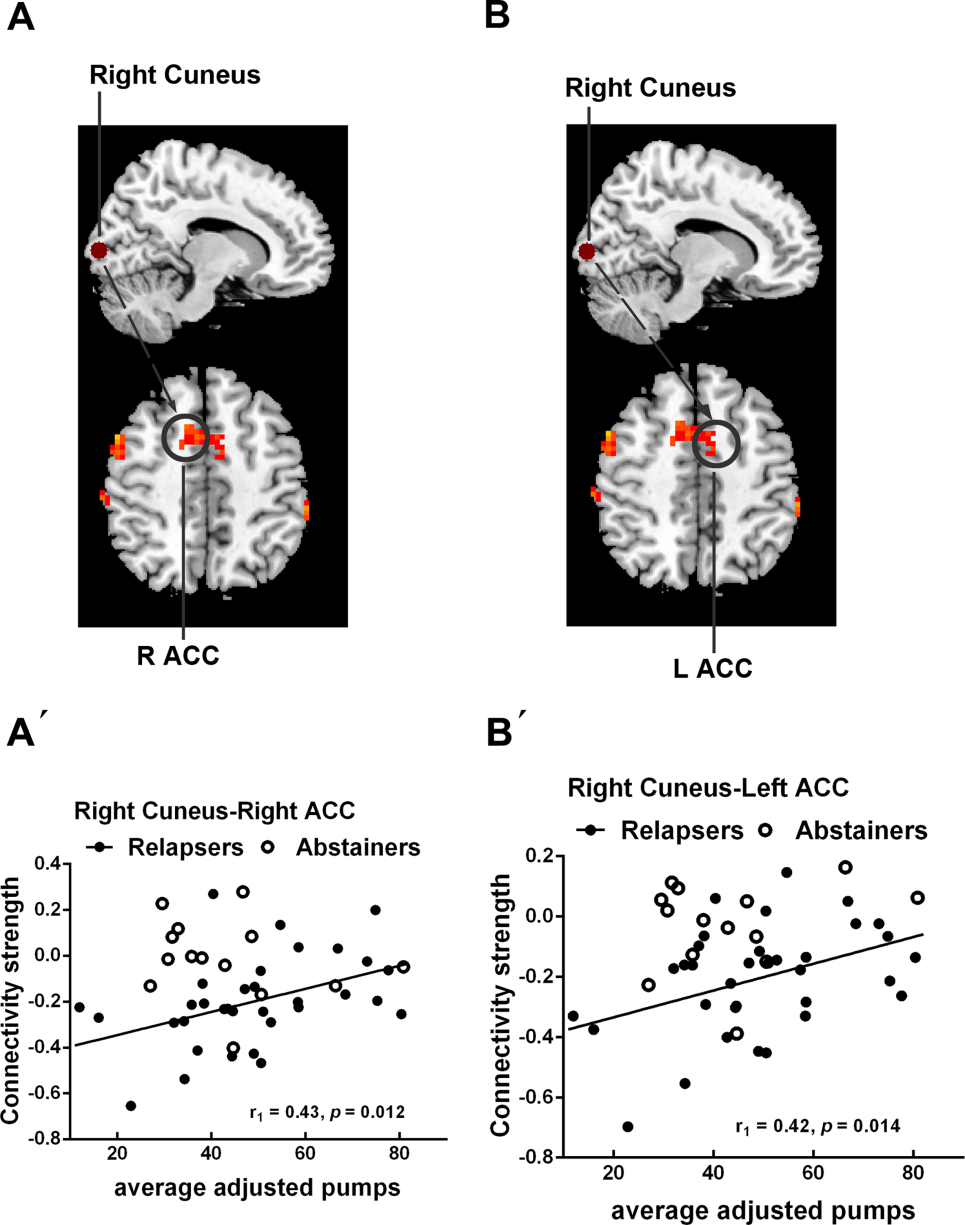
Correlations between MRI measures and behavioral measures of impulsivity in relapsers. (A´) The rsFC between the right cuneus and the right ACC showed significant positive correlation with average adjusted pumps in relapsers; (B´) The rsFC between the right cuneus and the left ACC showed significant positive correlation with average adjusted pumps in relapsers. (A-B) Brain maps of representative slices of related areas are also showed in the figure and colored dots represent their locations. Arrows are for illustrating purpose and do not imply directionality. Black dots and black line represent relapsers. Blank circles represent abstainers. Abbreviations: ACC, anterior cingulate cortex.

Correlation analysis was conducted to identify the relationship between MRI measures and behavioral measures of impulsivity in relapsers and abstainers. As shown in Fig 4, within relapsers, average adjusted pumps showed significant positive correlations with the connectivity strengths between the right cuneus and the bilateral ACC (*P* **<** 0.05, Bonferroni correction). Abstainers did not show any significant correlation.

## Discussion

### Main findings

The present study aimed to investigate GMV deficits and their potential downstream effects in rsFC during early stage of abstinence in ADPs associated with subsequent relapse outcomes and their implications for impulsivity. To our knowledge, this is the first study to report that both relapsers and abstainers exhibited significantly smaller GMV in the parietal-occipital regions relative to HCs in the context of alcohol dependence. Using these brain regions as seed regions, the rsFC analysis indicated that abstainers relative to relapsers demonstrated significantly decreased negative functional connectivity between the right cuneus and several regions, including the bilateral dlPFC, left insula, right premotor cortex and bilateral ACC. Regression analysis showed that abnormal GMV in the left cuneus and the rsFC between the right cuneus and the bilateral dlPFC after an average of 62 days abstinent could significantly predict relapse during the 90-day follow-up period. Additionally, the connectivity strength between the right cuneus and the bilateral ACC was positively correlated with average adjusted pumps.

### Reduced GMV in abstainers and relapsers

Our major results of VBM analysis suggested that abstainers only showed significantly reduced GMV in the cuneus relative to HCs; while relapsers showed significantly reduced GMV in more brain areas beyond the cuneus and regions surrounding the parieto-occipital sulcus. As we all know, reduced GMV in abstainers and relapsers can be explained by neurotoxic effects of alcohol and chronic alcohol abuse could cause the GM atrophy in alcohol-dependent individuals [25]. Many of the areas in which relapsers showed reduced GMV are consistent with previous studies which reported that alcohol-dependent individuals showed prominent GM reductions in the prefrontal cortex, the cingulate cortex, the thalamus and the hippocampus compared with HCs [25, 26]. The abstainers in our study did not show reduced GMV in the prefrontal cortex, striatum and hippocampus, though. A possible explanation is that GM abnormalities are less pronounced in abstainers after an average of 62 days abstinent, which may result in sustained remission from alcohol dependence. It has been shown that abstinence can lead to the recovery of GMV in some brain regions in alcohol-dependent individuals [27–29]. Furthermore, a recent study reported that significantly reduced GMV was observed in a region surrounding the parietal-occipital sulcus, which included the cuneus and precuneus in former heroin-dependent subjects who were drug free for more than 3 years [30]. Therefore, GM atrophy in the cuneus is not as easily reversible as other brain areas and may contribute to alcohol relapse. Another prospective study suggested that smaller GMV in the posterior-occipital was predictive of shorter time to relapse in alcohol-dependent subjects [15]. Therefore, the findings added the evidence that persistent GMV deficit in the posterior-occipital region is a predictive factor for the alcohol relapse. Given the fact that lower GMV in this region predicts alcohol relapse outcomes, it is important to examine its potential downstream effects in functional connectivity which may explain the impact of dysfunctional posterior-occipital circuit on the relapse to the alcohol use.

### rsFC differences between abstainers and relapsers

Our rsFC results indicated that relapsers demonstrated significantly increased negative functional connectivity strengths than abstainers between the right cuneus and several regions, including the bilateral dlPFC, left insula and bilateral ACC. These findings were in accordance with the compensation theory which was proposed in early studies of alcohol dependence. The cuneus hub is functionally connected to a visual network and is considered as a central architecture for the integration of visual information [31]. In fact, under normal condition, the visual network is negatively correlated with default mode network (DMN), which may contribute to maintain basic visual processing [31]. After healthy individuals performing higher-level cognitive task, the visual network becomes negatively correlated with the task-positive network, including the attention network, EC network and salience network, which may indicate an efficient computational state to facilitate independent task recruitment and switching during task performance [32–34]. However, a previous research has identified the pattern of expanded connectivity to regions outside the main networks in alcoholic compared with controls [35]. Specifically, during rest, alcoholic showed expanded and greater dlPFC connectivity with the cuneus than controls. For visual network, better task performance was related to expanded connectivity in alcoholic. The evidence suggested a mechanism to explain the EC and visual network impairments in alcoholic and supported the theory of network expansion as a neural mechanism for functional compensation [35]. This interpretation is also supported by another event-related fMRI study [36]. In this study, controls used basic visuospatial processes to perform a perceptual learning task, whereas detoxified alcoholic invoked higher-order cognitive processes (executive systems) to perform at normal levels. Therefore, Alcoholic may exert executive control to compensate for deficits in visual processing. In addition, the study suggested that the inefficient use of executive systems to accomplish relatively low-level cognitive task may leave little reserve of higher-level attentional capacity available if faced with additional cognitive demands [36]. According to this concept, when alcohol-dependent individuals face with motivationally visual stimuli, because of their inefficient strategies, the resources necessary to exert cognitive control may be unavailable, thus leading to early relapse to alcohol use. Taken together, our rsFC results confirm the compensation theory again. Furthermore, our finding that abnormal GMV in the cuneus and the cuneus-dlPFC connectivity after abstinence could significantly predict subsequent relapse is also consistent with this explanation.

### rsFC differences between HCs and relapsers

Another important finding of the rsFC analysis is that, relative to HCs, relapsers demonstrated significantly decreased positive functional connectivity between the left thalamus seed to the left inferior temporal gyrus and to the left parahippocampal gyrus, and between the right dlPFC seed to the left thalamus and to the left globus pallidus. These areas are extensively involved in attention, reward and emotion [31, 35, 37]. The decreased functional connectivity in relapsers may reflect diminished top-down cognitive control which is required to inhibit prepotent responses and indicate lack of attention when needed to arouse the awareness of the negative consequences of alcohol use [38–40].

### Associations between MRI measures and impulsivity

The elevated impulsivity in relapsers relative to HCs and abstainers found in the present study is consistent with previous reports [41, 42]. Our correlation analysis further demonstrated that higher impulsivity was associated with weaker negative connectivity between the right cuneus and the bilateral ACC in relapsers. Moreover, some correlative trends also indicate similar relationship between MRI measures and behavioral measures of impulsivity in relapsers and abstainers (see S2 Fig and S2 Text). These results also support the interpretation that relapsers may recruit expanded and greater functional network to maintain impulse control at relatively normal level. The ACC is repeatedly mentioned to be implicated in diverse functions including decision making, conflict monitoring and error detection and to serve as a critical neural substrate of impulsivity [38, 43–45]. Based on these findings, we speculate that alcohol-dependent individuals may still fail to flexibly regulate the cognitive control over drug reuse during extraneous motivational audiovisual stimuli (e.g., drug cues), indicating the role of impulsivity in relapse vulnerability. In future, further studies are needed to explore the relationship between impulsivity and relapse to alcohol use.

### Limitations

Some limitations in this study should be noted. Firstly, there were more smokers in relapsers and abstainers than in HCs and cigarettes use was not matched between the three groups. Although we can’t rule out the potential effects of smoking on the brain, the alcohol use may still be the dominant factor contributing to brain abnormalities according to the previous study [46]. Secondly, educational level was not matched between the three groups. However, we considered educational level as a covariate to avoid this confounding factor on any observed differences in the structure and function of the brain between groups when we performed the MRI analysis. Then, recent studies reported that the morphology of brains statistically differs between different origins (race) and Chinese brain template may be more suitable for Chinese populations [47, 48]. So, Chinese brain atlas is recommended to be applied to improve the performance in the neuroimaging studies involving Chinese populations. Furthermore, because no women were included in the current study, we were unable to examine sex-specific effects on structural and functional brain abnormalities. It is recommended that future studies evaluate sex differences in the relationship between brain abnormalities and relapse vulnerability, as there is some evidence showing greater sensitivity to alcohol neurotoxicity among women [49]. Finally, although the present study revealed the relationship between impulsivity and relapse to alcohol use, the specific neural mechanisms are needed to further explore, which could help to better understand the relapse process.

In summary, the present study demonstrated that ADPs which either later relapsed or abstained after achieving abstinence in the course of inpatient treatment had GM deficits in the parieto-occipital region. Relapsers also showed reduced GM volume in premotor and motor cortex, prefrontal cortex, cingulate cortex, thalamus, and cerebellum. Because of the GM deficits, we observed that patients who resumed alcohol intake had significantly increased cuneus-centered negative functional connectivity within a network of brain regions which are involved in executive control and salience when compared with those who achieved sustained abstinence. Present results provide evidence that the abnormal GMV in the cuneus and resting-state cuneus-prefrontal functional connectivity play an important role in early relapse to alcohol use and these MRI indices may serve as potential biomarkers that may identify individuals at high risk for early relapse.

## Supporting information

S1 Text Details about participants. (DOCX)

S2 Text Correlations between MRI measures and impulsivity in relapsers and abstainers. (DOCX)

S1 Table Grey matter volume and functional connectivity alterations in relapsers and abstainers. (DOCX)

S2 Table GMV reduction in relapsers versus HCs (FWE corrected for multiple comparisons across the entire volume). (DOCX)

S3 Table Summary of logistic regression analysis for different behavioral measure used as a predictor of early relapse. (DOCX)

S1 Fig Whole-brain GMV was compared between abstainers, relapsers and HCs. (DOCX)

S2 Fig Correlative trends between MRI measures and behavioral measures of impulsivity in abstainers and relapsers. (DOCX)
