# Supplementary material for: Combining gray matter volume in the cuneus and the cuneus-prefrontal connectivity may predict early relapse in abstinent alcohol-dependent patients
Source: PLoS One. 2018 May 7;13(5):e0196860. doi: 10.1371/journal.pone.0196860 (PMC5937790; doi:10.1371/journal.pone.0196860)
Supplement: S1 Text — (DOCX) [file pone.0196860.s001.docx]

**S1 Text.** Details about participants.

After enrollment in the study, ADPs have experienced serious physical, mental and social health issues, including alcohol withdrawal symptoms, family crisis, job loss and so on. Moreover, considering the effects on brain function, ADPs were excluded from the study if they had severe complications, including seizures and delirium tremens. During the inpatient treatment, ADPs received multidimensional therapeutic interventions for alcohol dependence, including medical detoxification, behavioral therapies and group counseling. All ADPs firstly underwent a acute detoxification process with oxazepam or diazepam. Oxazepam or diazepam dosage was individualized according to the severity of their withdrawal symptoms, including tremors, sweating and headache. Benzodiazepine dosage was gradually tapered and ADPs received a benzodiazepine prescription for no longer than two weeks. In addition to benzodiazepine, ADPs received thiamine (100 mg/day orally) and a multivitamin. ADPs were required to have at least 1 month (mean: 50.58 days) of abstinence before behavioral tests and MRI scans. When ADPs participated in the test and MRI scans, they received a multivitamin and were free of psychoactive medications for at least 1 week. After an average of 62 days abstinent (SD = 17.00), ADPs were discharged from the hospital. They continued to receive medication with antipsychotics, thiamine and multiple vitamin for outpatient treatment. ADPs were interviewed and asked whether they abstained or relapsed by telephone once every two weeks for a period of 3 months after discharge.
