# Supplementary material for: Combining gray matter volume in the cuneus and the cuneus-prefrontal connectivity may predict early relapse in abstinent alcohol-dependent patients
Source: PLoS One. 2018 May 7;13(5):e0196860. doi: 10.1371/journal.pone.0196860 (PMC5937790; doi:10.1371/journal.pone.0196860)
Supplement: S2 Text — (DOCX) [file pone.0196860.s002.docx]

**S2 Text.** Correlations between MRI measures and impulsivity in relapsers and abstainers.

Moreover, we also found some correlative trends between MRI measures and behavioral measures of impulsivity in relapsers and abstainers. Specifically, relapsers and abstainers both showed a significant negative trend between average adjusted pumps and the GMV of the right dlPFC (see S2 Fig A´). Relapsers showed a significant negative trend between the total score of the BIS-11 and the connectivity strength between the left thalamus and the left parahippocampal gyrus (see S2 Fig B´). Abstainers showed a significant positive trend between the total score of the BIS-11 and the connectivity strength between the right cuneus and the left ACC (see S2 Fig C´).
