# Supplementary material for: Combining gray matter volume in the cuneus and the cuneus-prefrontal connectivity may predict early relapse in abstinent alcohol-dependent patients
Source: PLoS One. 2018 May 7;13(5):e0196860. doi: 10.1371/journal.pone.0196860 (PMC5937790; doi:10.1371/journal.pone.0196860)
Supplement: S1 Table — (DOCX) [file pone.0196860.s003.docx]

**S1 Table.** Grey matter volume and functional connectivity alterations in relapsers and abstainers

| Seed | Brain regions | Side | Number of  voxels in cluster | *T* value | Peak MNI coordinates | | | | | | |
| --- | --- | --- | --- | --- | --- | --- | --- | --- | --- | --- | --- |
|  |  |  |  |  | X | | | Y | | Z | |
| **Grey matter volume** | | | | | | | | | | | |
|  | Abstainers < HCs |  |  |  |  | | |  | |  | |
|  | Cuneus | L | 128 | 5.69 | -8 | | | -96 | | -2 | |
|  | Abstainers > HCs |  |  |  |  | | |  | |  | |
|  | NS |  |  |  |  | | |  | |  | |
|  | Relapsers < HCs |  |  |  |  | | |  | |  | |
|  | Cuneus | L | 2137 | 6.72 | -9 | | | -73 | | 9 | |
|  |  | R | 191 | 5.81 | 12 | | | -93 | | 6 | |
|  | Precuneus | R | 2137 | 6.09 | 6 | | | -67 | | 22 | |
|  | Relapsers > HCs |  |  |  |  | |  | | |  | |
|  | NS |  |  |  |  | |  | | |  | |
| **Functional connectivity** | | | | | | | | | | | |
| Right Cuneus |  |  |  |  | |  | | |  | |  |
|  | Abstainers > Relapsers |  |  |  | |  | | |  | |  |
|  | dlPFC | L | 86 | 4.53 | | -36 | | | 39 | | 21 |
|  |  | R | 79 | 4.26 | | 54 | | | 9 | | 45 |
|  | Insula | L | 130 | 4.19 | | -39 | | | 12 | | 9 |
|  | Premotor cortex | R | 209 | 4.17 | | 9 | | | 9 | | 57 |
|  | ACC | L | 209 | 3.69 | | 12 | | | 15 | | 48 |
|  |  | R | 209 | 3.53 | | -6 | | | 9 | | 48 |
| Right Cuneus |  |  |  |  | |  | | |  | |  |
|  | HCs > Relapsers |  |  |  | |  | | |  | |  |
|  | dlPFC | R | 51 | 5.22 | | 54 | | | 0 | | 45 |
| Left Thalamus |  |  |  |  | |  | | |  | |  |
|  | HCs > Relapsers |  |  |  | |  | | |  | |  |
|  | ITG | L | 89 | 4.20 | | -42 | | | -30 | | -21 |
|  | ParaHip | L | 89 | 4.18 | | -27 | | | -12 | | -24 |
| Right dlPFC |  |  |  |  | |  | | |  | |  |
|  | HCs > Relapsers |  |  |  | |  | | |  | |  |
|  | Globus pallidus | L | 205 | 4.77 | | -12 | | | 6 | | 0 |
|  | Thalamus | L | 205 | 4.60 | | -6 | | | -15 | | 3 |

Abbreviations: R = Right, L = left. MNI: Montreal Neurological Institute; dlPFC: dorsolateral prefrontal cortex; ACC, anterior cingulate cortex; ITG, inferior temporal gyrus; ParaHip, parahippocampal gyrus; HCs: healthy control
