# Supplementary material for: Combining gray matter volume in the cuneus and the cuneus-prefrontal connectivity may predict early relapse in abstinent alcohol-dependent patients
Source: PLoS One. 2018 May 7;13(5):e0196860. doi: 10.1371/journal.pone.0196860 (PMC5937790; doi:10.1371/journal.pone.0196860)
Supplement: S2 Table — (DOCX) [file pone.0196860.s004.docx]

**S2 Table.** GMV reduction in relapsers versus HCs (FWE corrected for multiple comparisons across the entire volume)

| Brain regions | Side | Number of voxels in cluster | *T* value | Peak MNI coordinates | | |
| --- | --- | --- | --- | --- | --- | --- |
|  |  |  |  | X | Y | Z |
| Relapsers < HCs |  |  |  |  |  |  |
| Primary motor cortex | L | 362 | 6.65 | -45 | -19 | 37 |
| dPCC | R | 331 | 6.40 | 0 | -19 | 48 |
| Premotor cortex | R | 331 | 6.00 | 0 | -9 | 55 |
| dlPFC | R | 69 | 5.48 | 2 | 17 | 48 |
| Thalamus | L | 82 | 5.72 | -18 | -30 | -2 |
|  | R | 61 | 5.36 | 15 | -34 | 1 |
| Cerebellum | R | 60 | 5.41 | 3 | -37 | -17 |
| Relapsers > HCs |  |  |  |  |  |  |
| NS |  |  |  |  |  |  |

Abbreviations: R = Right, L = left. MNI: Montreal Neurological Institute; dPCC, dorsal posterior cingulate cortex; dlPFC: dorsolateral prefrontal cortex; HCs: healthy controls.
