# Supplementary material for: Combining gray matter volume in the cuneus and the cuneus-prefrontal connectivity may predict early relapse in abstinent alcohol-dependent patients
Source: PLoS One. 2018 May 7;13(5):e0196860. doi: 10.1371/journal.pone.0196860 (PMC5937790; doi:10.1371/journal.pone.0196860)
Supplement: S3 Table — (DOCX) [file pone.0196860.s005.docx]

**S3 Table.** Summary of logistic regression analysis for different behavioral measure used as a predictor of early relapse

| Predictor | B | SE B | Wald | *P* value |
| --- | --- | --- | --- | --- |
| Age | 0.10 | 0.063 | 2.54 | 0.11 |
| Education | 0.19 | 0.18 | 1.16 | 0.28 |
| Average dose of lifetime | 0.12 | 0.16 | 0.51 | 0.48 |
| Dose during peak use | -0.09 | 0.11 | 0.65 | 0.42 |
| Length of abstinence until MRI session | -0.016 | 0.017 | 0.95 | 0.33 |
| Current cigarettes per day | 0.018 | 0.038 | 0.22 | 0.64 |
| MAST scores | -0.21 | 0.11 | 3.44 | 0.064 |
| BIS-11 total scores | 0.17 | 0.14 | 1.42 | 0.23 |
| BIS-11 nonplanning scores | -0.24 | 0.20 | 1.42 | 0.23 |
| BIS-11 attention scores | -0.37 | 0.25 | 2.17 | 0.14 |
| HAMA scores | 0.34 | 0.31 | 1.16 | 0.28 |
| HAMD scores | -0.24 | 0.28 | 0.77 | 0.38 |
| Average adjusted pumps | -0.002 | 0.028 | 0.005 | 0.94 |

Note: Model coded 0 for Relapsers and 1 for Abstainers. Abbreviations: B, raw Beta coefficient; SE B, standard error for raw Beta coefficient; MAST, Michigan Alcoholism Screening Test; BIS-11, Barratt Impulsiveness Scale 11th version; HAMA, Hamilton Anxiety Scale; HAMD, Hamilton depressive Scale; MRI, magnetic resonance imaging.
