# Supplementary material for: Combining gray matter volume in the cuneus and the cuneus-prefrontal connectivity may predict early relapse in abstinent alcohol-dependent patients
Source: PLoS One. 2018 May 7;13(5):e0196860. doi: 10.1371/journal.pone.0196860 (PMC5937790; doi:10.1371/journal.pone.0196860)
Supplement: S1 Fig — (DOCX) [file pone.0196860.s006.docx]

**S1 Fig.** Whole-brain GMV was compared between abstainers, relapsers and HCs using SPM8 plus DAREL analysis. Decreased GMV also existed in several regions, including the right dPCC, the right premotor cortex, the left M1, the right dlPFC, the bilateral thalamus and the right cerebellum in Relapsers compared with HCs. One-way ANOVA was applied to test for group differences. A voxel level threshold was set at *P* < 0.05 (FWE-corrected). Abbreviations: dPCC, dorsal posterior cingulate cortex; M1, primary motor cortex; dlPFC, dorsolateral prefrontal cortex; GMV, grey matter volume.
