# Supplementary material for: Combining gray matter volume in the cuneus and the cuneus-prefrontal connectivity may predict early relapse in abstinent alcohol-dependent patients
Source: PLoS One. 2018 May 7;13(5):e0196860. doi: 10.1371/journal.pone.0196860 (PMC5937790; doi:10.1371/journal.pone.0196860)
Supplement: S2 Fig — (DOCX) [file pone.0196860.s007.docx]

**S2 Fig.** Correlative trends between MRI measures and behavioral measures of impulsivity in abstainers and relapsers. (A´) Relapsers and abstainers both showed a significant negative trend between average adjusted pumps and the GMV of the right dlPFC; (B´) Relapsers showed a significant negative trend between the total score of the BIS-11 and the connectivity strength between the left thalamus and the left parahippocampal gyrus; (C´) Abstainers showed a significant positive trend between the total score of the BIS-11 and the connectivity strength between the right cuneus and the left ACC. (A-C) Brain maps of representative slices of related areas are also showed in the figure and colored dots represent their locations. Arrows are for illustrating purpose and do not imply directionality. Black dots and black line represent relapsers. Blank circles and blue line represent abstainers. Abbreviations: dlPFC, dorsolateral prefrontal cortex; ParaHip, parahippocampal gyrus; TH, thalamus; ACC, anterior cingulate cortex; BIS, Barratt Impulsiveness Scale.
